# Study on the effect of community intervention on patients with hypertension in high-normal blood pressure

**DOI:** 10.3389/fmed.2026.1761106

**Published:** 2026-03-04

**Authors:** Qingxia Gao, Zhiguang Gao, Qianfeng Yang, Lijun Wang, Lishuang Xu

**Affiliations:** 1 Chengde Medical University, Chengde, China; 2 Chaoyang Central Hospital, Chaoyang, China; 3 Shengjing Hospital of China Medical University, Shenyang, China

**Keywords:** community intervention, health management, high-normal blood pressure, hypertension, lifestyle

## Abstract

**Introduction:**

Among patients with hypertension, maintaining blood pressure within the high-normal range is a common clinical scenario, often misinterpreted as adequate control. However, this level remains associated with cardiovascular risk and progression of target organ damage. Yet, evidence is lacking regarding effective community-based interventions in this population. This study aimed to evaluate the effects of a 12-month, community physician-led standardized health management intervention on blood pressure control rates, lifestyle improvements, and influencing factors in hypertensive patients with high-normal blood pressure, and to explore effective management strategies for this key population.

**Methods:**

A series of information surveys and health interventions were conducted among 721 patients (aged 18–80 years) with high-normal blood pressure from communities in Shenyang. Descriptive analysis and multivariable logistic regression analysis were used as primary analytical methods.

**Results:**

After 1 year of community intervention, both systolic and diastolic blood pressure significantly decreased in 721 individuals with high-normal blood pressure (*P* < 0.05). Knowledge about hypertension, awareness of prevention, medication adherence, and behavioral adherence also improved compared to pre-intervention levels (*P* < 0.05). A repeated-measures analysis of variance revealed a statistically significant main effect of time on both systolic and diastolic blood pressure [SBP: *F*_(1.90_, _1, 369.92)_ = 135.833, partial η^2^ = 0.159; DBP: *F*_(1.995_, _1, 436.10)_ = 50.181, partial η^2^ = 0.065, both *P* < 0.05]. Analysis of factors influencing blood pressure control at the end of the intervention demonstrated that poor medication adherence due to adverse drug reactions was associated with inadequate blood pressure control [OR (95% CI): 3.222 (1.169–8.878), *P* < 0.05]. Additionally, after the intervention, not reducing smoking was identified as a factor inversely associated with uncontrolled diastolic blood pressure [OR (95% CI): 0.192 (0.068–0.562), *P* < 0.05], while higher body weight remained associated with uncontrolled diastolic blood pressure [OR (95% CI): 1.018 (1.003–1.035), *P* < 0.05]. Furthermore, using non-drink-reducers as the reference group, alcohol reduction was identified as an influencing factor for uncontrolled systolic blood pressure [OR (95% CI): 2.550 (1.419–4.583), *P* < 0.05].

**Conclusion:**

Community-based interventions by primary care physicians targeting individuals with high-normal blood pressure can effectively modify unhealthy lifestyle habits and improve blood pressure control.

## 1 Introduction

The “2024 Chinese Guidelines for the Prevention and Treatment of Hypertension (Revised Edition)” defines high-normal blood pressure as systolic blood pressure ranging from 120 to 139 mmHg and/or diastolic blood pressure ranging from 80 to 89 mmHg (1). Although this stage typically lacks significant clinical symptoms, epidemiological and clinical studies suggest that this population already exhibits signs of early vascular and organ functional changes. Research indicates that high-normal blood pressure is associated with microvascular damage, atherosclerosis, and early renal function alterations (2). Zhen et al. (3) observed a tendency toward reduced carotid artery elasticity and impaired left ventricular diastolic function in individuals with high-normal blood pressure. Research data show that compared to the normal blood pressure group, the carotid-femoral pulse wave velocity is significantly elevated in the high-normal blood pressure group, suggesting that this population may have a higher propensity to atherosclerosis risk (4). Currently, evidence on long-term, comprehensive community-based interventions for this specific group remains scarce. This study conducted a 1-year intervention involving community healthcare workers to assess the effects on hypertension awareness and lifestyle modifications among community-dwelling individuals with hypertension and high-normal blood pressure, systematically evaluate its impact on blood pressure, and explore management strategies for this patient subgroup. The intervention outcomes for 721 community-based hypertension patients with high-normal blood pressure are reported as follows.

## 2 Materials and methods

### 2.1 Objective

This study aims to evaluate the effects of a 1-year, community-based standardized health management intervention program on blood pressure control rates, lifestyle improvements, and factors influencing blood pressure control among hypertensive individuals with high-normal blood pressure. This aim will be achieved through the following specific objectives: (1) to assess the change in systolic and diastolic blood pressure from baseline to 12-month post-intervention; (2) to evaluate changes in hypertension-related knowledge, attitudes, and self-reported practices (KAP) following the 12-month intervention; and (3) to identify factors associated with blood pressure control at the end of the intervention period.

### 2.2 Study design

This study uses a quasi-experimental pre-post intervention design. The study population comprised hypertensive patients with high-normal blood pressure, defined as individuals with a confirmed diagnosis of hypertension whose blood pressure remained within the high-normal range (systolic blood pressure 120–139 mmHg and/or diastolic blood pressure 80–89 mmHg) at follow-up. From January 2020 to December 2020, 721 patients completed the follow-up assessment. The effectiveness of the intervention was evaluated by comparing changes in the same group of participants before and after the implementation of the community doctor-led intervention. The study protocol was approved by the Medical Research Ethics Committee of the First Affiliated Hospital of China Medical University (reference number [2018]194). All participants provided informed consent.

### 2.3 Participants

In January 2020, recruitment was conducted in the community of Shenyang City, resulting in the enrollment of 3,791 patients through physical examination, blood pressure measurement, and questionnaire survey. Among them, 721 hypertensive individuals (19.02%) met the diagnostic criteria for high-normal blood pressure levels, with an average age of 67.38 ± 5.246 years, comprising 279 males (38.7%) and 442 females (61.3%).

#### 2.3.1 Inclusion criteria

Inclusion criteria included: (1) according to “the Chinese Guidelines for Hypertension Prevention and Treatment (2024 Revision),” the diagnostic criterion for high-normal blood pressure is defined as a systolic blood pressure (SBP) of 120–139 mmHg and/or a diastolic blood pressure (DBP) of 80–89 mmHg; (2) aged 18–80 years; (3) participants are required to willingly engage with community healthcare providers, possess effective communication skills, and have no previous history of cerebrovascular disorders; and (4) the blood pressure value was defined as the arithmetic mean of three consecutive measurements.

#### 2.3.2 Exclusion criteria

Exclusion criteria included: (1) individuals presenting with altered consciousness, mental health disorders, etc.; (2) those unable to engage in effective communication with community healthcare providers; and (3) participants with neurological conditions or infectious diseases.

### 2.4 Personal information

The demographic data of the cohort were collected using questionnaires, encompassing details such as name, gender, age, weight, height, community, telephone number, family history, history of hypertension, history of diabetes, and history of kidney disease. Additional information included their familiarity with hypertension diagnostic criteria, knowledge of daily salt intake guidelines for adults, awareness of hypertension prevention, changes in smoking, alcohol consumption, and salt intake, an increase in physical activity, and adjustments in medication due to adverse drug reactions.

### 2.5 Index definition

According to the “adult obesity feeding guide” (5), body mass index (BMI) = weight (kg)/height (m)^2^. BMI values are categorized as follows: BMI < 18.5 kg/m^2^ indicates low body mass, 18.5 kg/m^2^ ≤ BMI < 24 kg/m^2^ indicates normal weight, 24 kg/m^2^ ≤ BMI < 28 kg/m^2^ indicates overweight, and BMI ≥ 28 kg/m^2^ indicates obesity. The recommended daily salt intake for adults is ≤ 6 g. In this study, an average follow-up blood pressure of < 130/80 mmHg is defined as “controlled,” while an average follow-up blood pressure of ≥130/80 mmHg is defined as “uncontrolled.” Blood pressure control rate = number of blood pressure control/number of all high-normal hypertension patients × 100%. Patients provided subjective responses on factors such as alcohol consumption, smoking habits, salt intake, knowledge about hypertension, hypertension prevention measures, and medication adherence before and after the intervention.

### 2.6 Methods

#### 2.6.1 Assessment methods

Changes in sodium, alcohol, and tobacco intake were evaluated using self-reported data obtained from surveys conducted before and after the intervention. (1) Smoking: individuals who answered “yes” at baseline were defined as “current smokers.” “Smoking improvement” was defined as a change in status from a baseline smoker to “no” or “never” at follow-up; (2) alcohol consumption: individuals who answered “yes” at baseline were defined as “current drinkers.” “Alcohol consumption improvement” was defined as a change in status from a baseline drinker to “no” or “never” at follow-up; (3) increased physical activity: individuals who answered “yes” at baseline were defined as “engaging in half an hour of moderate-intensity exercise.” “Improvement in physical activity” was defined as a change from not meeting the half-hour exercise criterion at baseline to reporting “yes” at follow-up; and (4) reduced salt intake: individuals who answered “yes” at baseline were defined as “controlling salt intake.” “Improvement in salt reduction” was defined as a change from not controlling salt intake at baseline to reporting “yes” at follow-up. This study established three assessment time points: baseline, mid-intervention (Month 6), and post-intervention (Month 12). At baseline and post-intervention, key measurements included blood pressure, medication usage, and hypertension-related knowledge, attitudes, and practices, while the mid-intervention assessment focused on blood pressure and medication usage.

#### 2.6.2 Intervention methods

**Figure 1:**
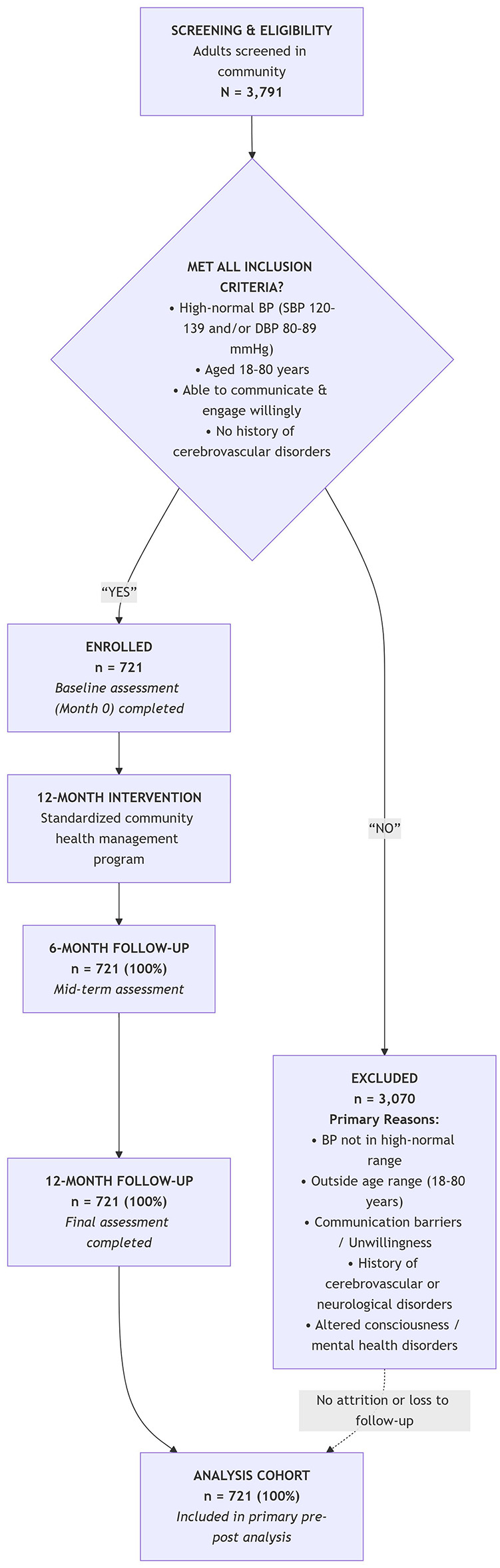
Flowchart of the intervention process.

(1) Community doctors were trained by cardiologists at China Medical University. The training includes blood pressure measurement, hypertension diagnosis, and preventive measures. These providers conducted regular patient follow-ups. In cases where patients were unable to attend scheduled appointments, community healthcare providers visited the patients' residences to ensure ongoing patient management; (2) hypertension knowledge dissemination for patients included information on salt intake, daily physical activity, hypertension diagnostic criteria, and preventive measures. This information was communicated through video presentations, face-to-face discussions by community doctors, display of hypertension knowledge posters, distribution of informational leaflets, organization of regular lectures, and preparation of educational brochures. These efforts aimed to raise awareness among patients about the cardiovascular risks associated with high-normal blood pressure and the impact of lifestyle modifications on blood pressure levels. Additionally, patients were advised to combine daily aerobic exercise (e.g., brisk walking, running, and cycling; >30 min) with active dietary management; and (3) community healthcare providers conducted scheduled weekly telephone follow-ups and semi-annual face-to-face consultation sessions with the patients. Utilizing telephone or text messaging, community physicians provided personalized guidance on medication adherence and lifestyle modifications while also addressing patient inquiries promptly. This strategy enhanced doctor–patient interaction and ensured intervention continuity, thereby improving overall management efficiency. Educating patients about the diverse health risks associated with hypertension, the cardiovascular disease risks linked to high-normal blood pressure, and the importance of non-pharmacological interventions. Facilitating enhanced doctor–patient communication and boosting management efficiency. A flowchart of the intervention protocol is presented in Figure 1.

#### 2.6.3 Measurement methods

Following the approval of the training materials, the affiliated community physicians underwent a 7-day training program led by cardiologists at the First Affiliated Hospital of China Medical University. This covered the detailed study protocol, management guidelines for high-normal blood pressure, key talking points for standardized lifestyle interventions (e.g., specific recommendations for salt reduction, physical activity, smoking cessation, and alcohol moderation), data collection procedures, and medical ethics requirements. In order to ensure the integrity of the data collected, it was essential that the investigator conducting the questionnaire used rigorous quality monitoring procedures. This entailed double-checking any data that might be questionable and subsequently reviewing it with another investigator. Finally, the data must be collated and entered in the appropriate format. All blood pressure measurements were taken at the brachial artery level. This study utilized standard office blood pressure measurement. Blood pressure was measured using a standard office measurement protocol. Follow-up visit schedules were jointly determined based on the availability of both the participants and the community health center. Participants rested for at least 5 min prior to measurement. A calibrated electronic sphygmomanometer (Omron 1200 U, Kyoto, Japan) was then used to measure blood pressure in the right upper arm. The entire process was supervised by a qualified community physician. Three consecutive readings were taken at 2-min intervals, and the average of these three readings was recorded as the participant's systolic and diastolic blood pressure during the follow-up visit. The physicians performing follow-up measurements were blinded to participants' prior blood pressure readings. Participants were instructed to abstain from caffeine consumption, vigorous exercise, and smoking for at least 30 min before measurement.

### 2.7 Statistical analysis

The data were analyzed by SPSS 27.0 (IBM Corporation, Armonk, NY, United States) software. Categorical data are presented as frequency (percentage). Systolic and diastolic blood pressure values before and after the intervention, which were non-normally distributed, are expressed using quartiles and were compared using the Wilcoxon signed-rank test. Knowledge, beliefs, and behavioral data measured before and after the intervention were compared using the χ^2^-test. Factors influencing the effectiveness of post-intervention blood pressure control were first assessed through univariate analysis, followed by multivariate analysis. Continuous data are presented as mean ± standard deviation (mean ± SD). Repeated measures analysis of variance (ANOVA) was used to assess the overall changes in blood pressure across three time points (pre-, mid-, and post-intervention). If the assumption of sphericity was not met (Mauchly's test of sphericity, *P* < 0.05), the Greenhouse–Geisser correction was applied. A *P*-value of < 0.05 was considered statistically significant.

## 3 Results

### 3.1 Baseline data

A total of 721 patients participated in the 1-year follow-up assessment. The mean age of participants was 67.38 ± 5.246 years. Among them, there were 279 males and 442 females. Within the cohort, 267 individuals were aged 65 years or younger, comprising 37% of the sample, while 454 individuals were between 66 and 80 years old, accounting for 63% of the total. The distribution of participants based on body mass index categories was as follows: five individuals (0.7%) had low BMI, 221 individuals (30.7%) had normal BMI, 357 individuals (49.5%) were classified as overweight, and 138 individuals (19.1%) were categorized as obese. A prevalence of 0.4% was observed for comorbid kidney disease, while 99.6% of individuals exhibited no comorbid kidney disease. A total of 21.8% of the population exhibited comorbid diabetes, while 78.2% demonstrated non-comorbid diabetes. The study population included 68.5% never-smokers and 31.5% current smokers, with corresponding proportions of non-drinkers and current drinkers being 67.7 and 32.3%, respectively (Table 1).

**Table 1:** Baseline characteristics of individuals with high-normal blood pressure.

**Items**	**Divide into groups**	**Number (*n*)**	**Component ratio (%)**
Gender	Male	279	38.7
	Female	442	61.3
Age	≤65	267	37
	65–80	454	63
BMI	Low body weight	5	0.7
	Normality	221	30.7
	Overweight	357	49.5
	Obesity	138	19.1
Whether hypertension was combined with diabetes	Yes	157	21.8
	No	564	78.2
Whether hypertension was combined with renal disease	Yes	3	0.4
	No	718	99.6
Whether you smoke or not	Yes	227	31.5
	No	494	68.5
Whether you drink or not	Yes	233	32.3
	No	488	67.7
Medications used	ACEI	28	3.9
	ARB	128	17.8
	CCB	273	37.9
	Diuretic	13	1.8
	Beta-adrenergic blocking agents	25	3.5
	Other drug classes	18	2.5

### 3.2 Relevant conditions of patients with high-normal hypertension before and after intervention

There was no statistically significant difference in medication usage among the participants before and after the intervention, as shown in Table 2.

**Table 2:** Medication before and after intervention in patients with high-normal blood pressure.

**Items**	**Pre-intervention**		**Post-intervention**		**χ^2^-value**	***P*-value**	**OR and 95% CI**
	**Number of samples**	**Percentages (%)**	**Number of samples**	**Percentages (%)**			
No medication	277	38.4	276	38.3	0.003	0.957	0.994 (0.804–1.229)
One kind of drug	411	57.0	407	56.4	0.045	0.873	0.978 (0.794–1.204)
Two or more drugs	33	4.6	38	5.3	0.370	0.627	1.160 (0.719–1.871)

#### 3.2.1 Changes in blood pressure before and after intervention

Following the intervention, there was a statistically significant improvement in the blood pressure values of the patients (*Z* = −7.309, −6.392, *P* < 0.05). The blood pressure control rate increased from 19.7% (*n* = 142) at baseline to 36.3% (*n* = 262) post-intervention. After intervention, the number of individuals based on the systolic blood pressure categories was as follows: 138 individuals (19.1%) had systolic blood pressure < 120 mmHg, 516 individuals (71.6%) had systolic blood pressure between 120 and 139 mmHg, and 67 individuals (9.3%) had systolic blood pressure ≥140 mmHg. Regarding diastolic blood pressure levels after the intervention, 486 individuals (67.4%) had diastolic blood pressure < 80 mmHg, 225 individuals (31.2%) had diastolic blood pressure between 80 and 89 mmHg, and 10 individuals (1.4%) had diastolic blood pressure ≥90 mmHg, as detailed in Table 3.

**Table 3:** Blood pressure before and after intervention in patients with high-normal blood pressure (*P*_25,75_, mmHg).

**Item**	**Pre- intervention**		**Post- intervention**		***Z*-value**	***P*-value**
	**Median**	**IQR**	**Median**	**IQR**		
SBP	132	(128,136)	130	(121,135)	−7.309	<0.01
DBP	78	(71,82)	76	(70,80)	−6.392	<0.01

#### 3.2.2 Comparison of systolic and diastolic blood pressure at pre-, mid-, and post-intervention time points

Repeated-measures analysis of variance indicated that both systolic and diastolic blood pressure changed significantly across the three time points (pre-, mid-, and post-intervention) (systolic: *F* = 135.83, partial η^2^ = 0.159, *P* < 0.01; diastolic: *F* = 50.18, partial η^2^ = 0.065, *P* < 0.01), as depicted in Table 4.

**Table 4:** Comparison of systolic and diastolic blood pressure at pre-, mid-, and post-intervention time points.

**Variable**	**Time**	**Mean ±SD (mmHg)**	**Within-subjects test**		
			***F*** **(df)**	***P*** **-value**	**Partial** η^2^
SBP	Pre-intervention	131.70 ± 5.10	135.833 (1.90, 1,369.92)	<0.01	0.159
	Mid-intervention	136.69 ± 13.10			
	Post-intervention	129.08 ± 11.24			
DBP	Pre-intervention	76.74 ± 7.55	50.181 (1.995, 1,436.10)	<0.01	0.065
	Mid-intervention	78.00 ± 9.10			
	Post-intervention	74.79 ± 8.15			

#### 3.2.3 KAP model and systolic blood pressure control

After the intervention of community doctors, statistically significant associations were observed between changes in systolic blood pressure and increased physical activity, reduced salt intake, decreased smoking, reduced alcohol consumption, awareness of hypertension diagnosis, awareness of hypertension prevention, and improved drug compliance to manage adverse reactions (χ^2^ = 13.799, 14.542, 5.511, 10.078, 27.362, 11.931, 6.238, all *P* < 0.05). However, there were no significant differences identified in associations with forgetting to take medications or awareness of daily salt intake guidelines for adults, as depicted in Table 5.

**Table 5:** Effect of a community-based intervention model on systolic blood pressure in individuals with high-normal blood pressure.

**Post-intervention**	**Whether or not to improve**	**Systolic blood pressure after intervention (number)**		**χ^2^-value**	***P*-value**	**OR and 95% CI**
		≥**130 mmHg**	<**130 mmHg**			
Whether they increased physical labor	Yes	390	276	13.799	<0.001	0.344 (0.192–0.167)
	No	18	37			
Whether they reduced salt intake	Yes	382	266	14.542	<0.001	0.385 (0.233–0.638)
	No	26	47			
Whether they reduced smoking	Yes	92	88	5.511	0.019	0.448 (0.227–0.884)
	No	15	32			
Whether they reduced drinking	Yes	80	77	10.078	0.002	0.392 (0.218–0.705)
	No	22	54			
Whether they had poor adherence to medication due to adverse reactions	Yes	5	13	6.238	0.013	3.493 (1.232–9.903)
	No	403	300			
Whether they had poor adherence to medication due to forgetfulness	Yes	67	55	0.167	0.683	1.085 (0.733–1.605)
	No	341	258			
Whether to know the daily salt intake of adults	Yes	395	296	2.239	0.135	0.573 (0.274–1.198)
	No	13	17			
Whether they mastered the diagnosis of hypertension	Yes	389	262	27.362	<0.001	0.251 (0.145–0.435)
	No	19	51			
Whether they had awareness of hypertension prevention	Yes	395	284	11.931	<0.001	0.322 (0.165–0.631)
	No	13	29			

#### 3.2.4 KAP model for diastolic blood pressure control

The analysis indicates that reducing smoking has a statistically significant impact on diastolic blood pressure (χ^2^ = 11.746, *P* < 0.05), whereas other factors (such as increasing physical activity and reducing salt intake) show no significant association, as detailed in Table 6.

**Table 6:** Effect of a community-based intervention model on diastolic blood pressure in individuals with high-normal blood pressure.

**Post-intervention**	**Whether or not to improve**	**Diastolic blood pressure after intervention (number)**		**χ^2^-value**	***P*-value**	**OR and 95% CI**
		≥**80 mmHg**	<**80 mmHg**			
Whether they increased physical labor	Yes	222	444	2.174	0.140	0.619 (0.326–1.177)
	No	13	42			
Whether they reduced salt intake	Yes	216	432	1.594	0.207	0.704 (0.407–1.217)
	No	19	54			
Whether they reduced smoking	Yes	61	119	11.746	<0.001	0.181 (0.062–0.529)
	No	4	43			
Whether they reduced drinking	Yes	50	107	2.246	0.134	0.617 (0.327–1.164)
	No	17	59			
Whether they had poor adherence to medication due to adverse reactions	Yes	3	15	2.131	0.144	2.463 (0.706–8.592)
	No	232	471			
Whether they had poor adherence to medication due to forgetfulness	Yes	40	82	0.002	0.960	0.989 (0.653–1.498)
	No	195	404			
Whether they mastered the diagnosis of hypertension	Yes	219	432	3.345	0.067	0.584 (0.327–1.695)
	No	16	54			
Whether to know the daily salt intake of adults	Yes	227	464	0.501	0.479	0.743 (0.326–1.695)
	No	8	22			

#### 3.2.5 Changes in KAP pre- and post-intervention

A comparison of the KAP of the unified community physician intervention was conducted before and after the intervention. The results demonstrated a significant increase in awareness of hypertension-related knowledge and prevention, medication, and behavioral adherence following the implementation of the intervention when compared with the pre-intervention period. The difference was statistically significant, as outlined in Table 7.

**Table 7:** Changes in KAP pre- and post-intervention.

**Lifestyle**	**Pre-intervention *n* (%)**	**Post-intervention *n* (%)**	**χ^2^-value**	***P*-value**	**OR and 95% CI**
**Knowledge and awareness of hypertension prevention**					
Mastering the diagnostic criteria of hypertension	449 (62.3%)	651 (90.3%)	156.401	<0.01	5.634 (4.220–7.521)
Awareness of hypertension prevention	472 (65.5%)	679 (94.2%)	184.475	<0.01	8.529 (6.026–12.071)
Mastering daily salt consumption	450 (62.4%)	691 (95.8%)	243.864	<0.01	13.871 (9.341–20.598)
**Medication and behavioral adherence**					
Poor medication adherence due to forgetfulness	233 (32.3%)	122 (16.9%)	46.042	<0.01	0.427 (0.332–0.547)
Medication adherence due to adverse reaction	79 (11.0%)	18 (2.5%)	41.127	<0.01	0.208 (0.123–0.351)
Smoking reduction	104 (14.4%)	180 (25%)	54.314	<0.01	4.529 (2.995–6.851)
Alcohol reduction	65 (9.0%)	157 (21.8%)	72.815	<0.01	5.339 (3.593–7.935)
Salt intake reduction	277 (38.4%)	648 (89.9%)	415.031	<0.01	14.228 (10.703–18.915)
Increased physical activity	315 (43.7%)	666 (92.4%)	392.834	<0.01	15.607 (11.426–21.319)

### 3.3 Predictors of blood pressure control following intervention in high-normal hypertension

In the post-intervention analysis, blood pressure control was designated as the dependent variable, categorized as controlled (assigned a value of 1) and uncontrolled (assigned a value of 0). Blood pressure control is defined as systolic blood pressure <130 mmHg and diastolic blood pressure <80 mmHg. In the univariate analysis, the following factors demonstrated statistical significance and were included as independent variables in the subsequent multivariate model: poor medication adherence due to adverse reactions, knowledge of hypertension diagnostic criteria, reduction in smoking, reduction in alcohol consumption, reduction in dietary salt intake, and increase in physical labor. Multiple logistic regression analyses were used. The results showed that poor adherence to medication due to adverse reactions is a risk factor for controlled blood pressure (*P* < 0.05), as outlined in Table 8.

**Table 8:** Factors associated with blood pressure control at the end of intervention.

**Variables**	***B***	***SE***	**Wald-value**	***P*-value**	**OR-value and 95% CI**
**Whether they had poor adherence to medication due to adverse reactions**					
No					1.00
Yes	1.170	0.517	5.118	0.024	3.222 (1.169–8.878)

#### 3.3.1 Factors influencing diastolic blood pressure after intervention

In the post-intervention analysis, diastolic blood pressure was defined as the dependent variable, with diastolic blood pressure ≥80 mmHg assigned a value of 1 and diastolic blood pressure <80 mmHg assigned a value of 0. Univariate analysis identified the following factors with statistical significance: smoking reduction status, awareness of hypertension diagnostic criteria, alcohol reduction status, gender, and body weight. These significant factors were subsequently included as independent variables in a multivariate regression analysis. The results demonstrated that failure to reduce smoking and higher body weight were significant factors influencing uncontrolled diastolic blood pressure (*P* < 0.05), as outlined in Table 9.

**Table 9:** Analysis of influencing factors of diastolic blood pressure ≥80 mmHg in patients with high-normal blood pressure at the end of intervention.

**Variables**	***B***	***SE***	**Wald-value**	***P*-value**	**OR-value and 95% CI**
**Whether to reduce smoking**					
Yes			10.560	0.005	1.00
No	−1.650	0.547	9.083	0.003	0.192 (0.068–0.562)
Never	0.078	0.186	0.177	0.674	1.082 (0.751–1.558)
Weight	0.018	0.008	5.245	0.022	1.018 (1.003–1.035)

#### 3.3.2 Factors influencing systolic blood pressure after intervention

After the intervention, systolic blood pressure was defined as the dependent variable, with systolic blood pressure ≥130 mmHg designated as 1 and systolic blood pressure <30 mmHg as 0. In the univariate analysis, factors such as body weight, mastering daily salt consumption, smoking reduction, alcohol reduction, dietary salt reduction, and increased physical activity were statistically significant. Multiple logistic regression analysis revealed that reducing drinking was identified as an independent factor significantly influencing uncontrolled systolic blood pressure (*P* < 0.05), as outlined in Table 10.

**Table 10:** Analysis of influencing factors of systolic blood pressure ≥130 mmHg in patients with high-normal blood pressure at the end of intervention.

**Variables**	***B***	***SE***	**Wald-value**	***P*-value**	**OR-value and 95% CI**
**Whether they reduced drinking**					
No					1.00
Yes	0.936	0.299	12.604	0.003	2.550 (1.419–4.583)

This analysis was restricted to participants who reported alcohol consumption at baseline (n = 233), with “never drinkers” excluded from the sample.

## 4 Discussion

This study confirms that a community physician-led intervention based on the Knowledge-Attitude-Practice (KAP) model can improve blood pressure control and promote the adoption of healthy lifestyles among individuals with high-normal blood pressure. Specifically, the intervention not only significantly reduced both systolic and diastolic blood pressure and substantially increased the blood pressure control rate but also concurrently improved patients' knowledge about hypertension, health beliefs, and key behaviors such as smoking cessation, alcohol restriction, and physical activity. These findings demonstrate the effectiveness of this community-based intervention model for this specific population.

Abundant evidence indicates that interventions based on the KAP model delivered by community physicians can improve both blood pressure control and health behaviors. On the one hand, individuals with high-normal blood pressure face an elevated risk of cardiovascular events (6, 7). On the other hand, studies have shown that even modest reductions in blood pressure within this population can lead to cardiovascular benefits (8, 9). Liu et al. (10) found that among 607 prehypertensive individuals who received lifestyle interventions, 329 returned to normal blood pressure, 244 remained prehypertensive, and 34 progressed to hypertension. This demonstrates the necessity of community-based interventions and provides a research foundation for the present study.

This study demonstrates that the Knowledge-Attitude-Practice (KAP) model led by community physicians is effective in improving blood pressure control. Consistent with the findings of the intervention study by Jafar et al. (11), our intervention achieved significant reductions in blood pressure and a substantial increase in the blood pressure control rate (from 19.7 to 36.3%). The core clinical significance of this result lies in its validation of the effectiveness of community-based interventions. Previous evidence indicates that for every 5 mm Hg reduction in systolic blood pressure, the risk of cardiovascular events decreases by approximately 10% (12). This provides strong support for the expected effectiveness of implementing such structured behavioral intervention models at the community level.

This study confirms that an intervention based on the knowledge-attitude-practice model can improve the knowledge level, preventive awareness, and behavioral habits of individuals with high-normal blood pressure. It also helps mitigate poor medication adherence caused by forgetfulness and adverse drug reactions. These improvements in cognition and behavior contribute to better blood pressure control. This finding is consistent with the results of the PREMIER trial (13), which demonstrated that lifestyle improvements can reduce blood pressure and lower the risk of cardiovascular disease.

Multivariate analysis indicated that adverse drug reactions are an independent risk factor for uncontrolled blood pressure. This finding highlights a limitation in current hypertension management models: even with effective community-based behavioral interventions, medication side effects remain a key clinical barrier to treatment continuity. The rapid decline in medication adherence observed by He et al. (14) after hospital discharge corroborates the significant impact of adverse drug reactions identified in this study. Both findings collectively suggest that patients' subjective experiences influence medication adherence, thereby compromising the effectiveness of drug therapy. Therefore, future community management should place greater emphasis on medication adherence and provide feasible alternative treatment options when patients discontinue or reduce their medication in order to achieve effective blood pressure control.

The statistical analysis of this study indicated a negative correlation between not reducing smoking and uncontrolled diastolic blood pressure, a finding that contradicts both the widely accepted consensus that smoking elevates blood pressure (15, 16) and the expectations of this study. In contrast, weight gain was positively correlated with uncontrolled diastolic blood pressure. Research (17) has shown that smokers have a higher incidence of carotid artery plaques and increased microcalcification. We believe that this paradoxical finding may stem from uncontrolled confounding biases in the study, such as the possibility that patients who continued smoking received more intensive antihypertensive treatment. Additionally, recall bias and social desirability bias may have influenced patients' self-reported smoking status, and the smaller sample size in the non-smoking reduction group may also have contributed to the discrepancy from expectations. This is consistent with the findings of Liu et al. (18), whose study similarly observed the paradoxical phenomenon of lower average diastolic blood pressure in smokers compared to non-smokers. The authors noted that the underlying mechanism of this phenomenon remains unclear and cautioned against inferring causality. Therefore, future research should adopt more comprehensive measures to control for confounding factors, such as collecting more detailed treatment plans and patient adherence data and increasing the sample size to accurately clarify the true relationship between smoking cessation and blood pressure control.

A study by Qi et al. (19) found that drinkers had significantly higher systolic and diastolic blood pressure than non-drinkers, and that long-term heavy alcohol consumption adversely affects blood pressure. Therefore, reducing alcohol intake is generally considered an important measure for improving blood pressure control. However, in this study, logistic regression analysis revealed that “reducing alcohol consumption” was a risk factor for uncontrolled systolic blood pressure. This contradictory statistical finding is entirely inconsistent with expectations. Upon deeper reflection, we believe the reason for this result may lie in the fact that the relevant information in this study was collected through questionnaires, which may have introduced self-reporting bias to the data. Additionally, the study population was older, making it more susceptible to recall bias. Consequently, this result cannot be interpreted as a causal relationship but rather only as an association between reduced alcohol consumption and uncontrolled systolic blood pressure. To verify whether a causal relationship exists, controlled experiments should be conducted in future studies.

In conclusion, the Knowledge-Attitude-Practice (KAP) intervention model delivered by community physicians demonstrated significant effectiveness in individuals with hypertension at high-normal blood pressure levels, leading to substantial improvements in lifestyle behaviors and enhanced blood pressure control rates in this population.

It should be noted that the study was subject to the following limitations: (1) reliance on self-reported lifestyle data is susceptible to bias, potentially overestimating the improvements; (2) only a single-arm BP measurement at initial assessment may compromise the accuracy of baseline data; (3) given that this study uses a single-group pre-post design without a parallel control group, the observed improvements in outcomes such as blood pressure control, while statistically significant, have limitations in causal inference. Terms such as “improvement” used in this paper are primarily based on statistical comparisons of indicators before and after the intervention. Future randomized controlled trials are still required to further validate the causal relationship between the intervention measures and the outcomes; (4) the study population consisted of hypertensive patients whose blood pressure was maintained at high-normal levels through treatment; and (5) this study did not report cardiovascular disease status, as these data were not collected.

## Data Availability

The raw data supporting the conclusions of this article will be made available by the authors, without undue reservation.
